# Overwhelming response to Dabrafenib in a patient with double BRAF mutation (V600E; V600M) metastatic malignant melanoma

**DOI:** 10.1186/1756-8722-5-60

**Published:** 2012-10-02

**Authors:** Giovanni Ponti, Aldo Tomasi, Giovanni Pellacani

**Affiliations:** 1 Department of Clinical and Diagnostic Medicine and Public Health, University Hospital of Modena and Reggio Emilia, via del Pozzo 7, 41100, Modena, Italy; 2 Department of Head and Neck Surgery, Division of Dermatology, University Hospital of Modena and Reggio Emilia, Modena, Italy

## Abstract

The recent findings brought the necessity of testing the mutational status of a series of genes which had been already identified as responsible for melanomas development and progression, such as BRAF, CKIT and PTEN: the consequent results are, in fact, essential to guide the assessment of the novel treatment protocols based on tailored targeted therapies. We present here the case of a 66 year-old male patient, diagnosed with an advanced melanoma in June 2011, and treated with Dabrafenib for double mutant metastatic disease. The patient was referred to our attention for a large exophytic malignant melanoma on the left shoulder. After complete surgical excision and elective lymph node dissection for presence of metastatic sentinel lymph node, the patient has started high-dose interferon alfa-2b injections as adjuvant therapy for a complete negative staging. The treatment was interrupted in August 2011 due to the appearance of metastatic lymph nodes. Tumor burden was rapidly growing reaching in few months the size of a tennis ball for the tumor mass located in the shoulder. Mutational study of the tumor revealed a double BRAF mutation on V-600E and V600M. This finding incited us to enroll the patient in compassionate Dabrafenib clinical trial. The therapy was started on may 2012 at 150 mg bid dosage. Almost surprisingly for the rapidity of the effect, one week later the lesion on the shoulder has reduced its size by 60% and one month later it has completely disappeared from sight. CT scan of June 2012 documented the astonishing clinical response.

## 

The last year has finally experienced a real breakthrough in advanced melanoma therapy, that had probably been awaited for decades: molecular targeted therapies have been added to old and often impotent treatments in the battle against its metastatic disease.

It is nowadays well known that prognosis is dramatically influenced by primary tumor stage and chanches to be cured are very low in presence of non-operable metastatic disease. In fact, metastatic melanoma faces a poor outcome, with a predicted 5-year survival rate less than 10%, according to the metastases site and serum LDH levels [1].

The recent findings brought the necessity of testing the mutational status of a series of genes which had been already identified as responsible for melanoma development and progression, such as BRAF, CKIT and PTEN (MEK and mTOR pathways): the consequent results are, in fact, essential to guide the assessment of the novel treatment protocols based on tailored targeted therapies. Among them, Vemurafenib ((RG 7204; Roche, Basel, Switzerland) and Dabrafenib (GSK2118436), are the selective inhibitors of BRAF kinase activity that competitively inhibit ATP [2,3], suppressing a downstream pharmacodynamic biomarker (pERK) in tumour cell lines. In particular, the drug has demonstrated an antiproliferative activity against multiple BRAF mutant tumour cell lines and achieved biomarker suppression and tumour regression in BRAF mutant xenograft models.

At this regard we present here the case of a 66 year-old male patient, diagnosed with an advanced melanoma in June 2011, and treated with Dabrafenib for double mutant metastatic disease.

The patient was referred to our attention for a 5 cm large exophytic skin lesion on the left shoulder, that revealed to be a malignant melanoma (Figure 1a). After complete surgical excision and elective lymph node dissection for presence of metastatic sentinel lymph node, the patient has started high-dose interferon alfa-2b injections (Intron) as adjuvant therapy for a complete negative staging. The treatment was interrupted in August 2011 due to the appearance of left axillary and brachial nodular masses, that appeared as metastatic lymph nodes at CT scan in February 2012. Tumor burden was rapidly growing reaching in few months the size of a tennis ball for the tumor mass located in the shoulder that determined the uncomfortable “hump” on the patient’s back vz(Figure 1b).

**Figure 1:**
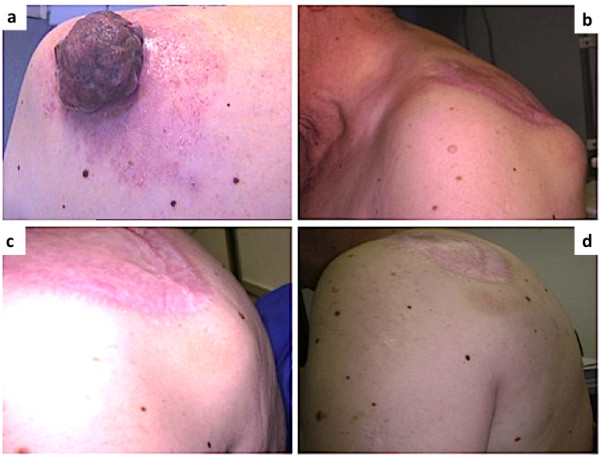
**Advanced cutaneous malignant melanoma.** Large exophytic skin melanoma on the left shoulder (Panel **a**); Left axillary and brachial metastatic masses (Panel **b**); reduction of the shoulder metastatic lesions by 60% after 1 week of Dabrafenib therapy (Panel **c**); Melanoma metastatic lesions completely disappeared after less than 1 month of therapy (Panel **d**)

Mutational study of the tumor revealed a double BRAF mutation on V-600E and V600M. Preliminary evidences showed an antiprolifetive activity of Dabrafenib also on rare BRAF somatic mutations different from the V600E [4,5]. This finding incited us enroll the patient in compassionate Dabrafenib clinical trial. The therapy was starded on may 2012 at 150 md bid dosage. Almost surprisingly for the rapidity of the effect, one week later the lesion on the shoulder has reduced its size by 60% (Figure 1c) and one month later it has completely disappeared from sight (Figure 1d). CT scan of June 2012 documented the astonishing clinical response. The patients did not experience any adverse reactions except for nausea in the first few days of therapy.

Obviously, the therapeutic benefit improved patient quality of life and mood. Based on previous experiences in the use of anti-BRAF molecules we cannot be optimistic and a relapse of the disease is expected at any time, so the patient is nowadays in constant monitoring. While we eagerly keep monitoring the evolution of our patient’s disease, we are awaiting the new advances in melanoma targeted therapy. Molecular targeted therapies (e.g. B-RAF inhibitors) have reached high response rates but unfortunately rather short response duration (Progression Free Survival of 6 months), while Immunotherapy showed slower but more durable results [6]. We expect promising outcomes from both the multi-target molecular therapy and the combination of molecular targeted therapies and Immunotherapy that should provide a long lasting outcome together with high response rates [7]. Moreover, there are still many ongoing trials both in mono-therapy and in combination with GSK1120212, a MEK inhibitor, that can greatly strengthen our hopes for a new era in melanoma treatment.

It is necessary to understand and overcome the limitations of these strategies, especially those concerning resistance mechanisms, in order to transform ephemeral remissions in long-lasting healing.

## Consent

Written informed consent was obtained from the patient for publication of this case report and accompanying images.

## Competing interests

The authors declare that they have no competing interests.

## Authors' contributions

GP was responsible of the clinical management of the patient, acquisition of data, drafting the manuscript; AT was supervisor of management and interpretation of data; GP was responsible of the scientific revision, discussion and editing of the manuscript. All authors read and approved the final manuscript.
